# Empathic embarrassment towards non-human agents in virtual environments

**DOI:** 10.1038/s41598-023-41042-3

**Published:** 2023-09-12

**Authors:** Harin Hapuarachchi, Kento Higashihata, Maruta Sugiura, Atsushi Sato, Shoji Itakura, Michiteru Kitazaki

**Affiliations:** 1 Department of Computer Science and Engineering, Toyohashi University of Technology, Toyohashi, Aichi Japan; 2 Faculty of Humanities, University of Toyama, Toyama, Japan; 3 Center for Baby Science, Doshisha University, Kyoto, Japan

**Keywords:** Empathy, Social behaviour

## Abstract

Humans feel empathic embarrassment by witnessing others go through embarrassing situations. We examined whether we feel such empathic embarrassment even with robot avatars. Participants observed a human avatar and a robot avatar face a series of embarrassing and non-embarrassing scenarios. We collected data for their empathic embarrassment and the cognitive empathy on a 7-point Likert scale. Both empathic embarrassment and cognitive empathy were significantly higher in the embarrassed condition compared to the non-embarrassed condition with both avatars, and the cognitive empathy was significantly higher with the human avatar. There was a tendency of participants showing a higher level of skin conductance while watching the human avatar go through embarrassing situations compared to the robot avatar. A following experiment showed that the average plausibility of the embarrassed condition was significantly higher with the human avatar compared to the robot avatar. However, plausibility scores for emotion were not significantly different among the conditions. These results suggest that humans can feel empathic embarrassment as well as cognitive empathy for robot avatars while cognitive empathy for robot avatars is comparatively lower, and that part of the empathic difference between human and robot avatars might be due to the difference of their plausibility.

## Introduction

Empathy is a factor that is long considered to be a mediator or a contributor to positive human interactions, altruistic behavior and sympathy^1^. The term “empathy” has been used as a label for a number of psychological processes and related consequences over the past century^2^. One of the most common definitions of empathy is “feeling a vicarious emotion that is congruent with but not necessarily identical to the emotion of another”^3^. Empathy is distinguishable since it is felt on behalf of another person, whereas other emotions are generally felt on one’s own behalf^4^. Even though it is also possible to respond with empathic joy when another is experiencing positive states^5^, empathy is often interpreted as an emotion felt by witnessing another’s suffering^2,6,7^, pain^8–11^, anger caused by injustice^6,12,13^, or even embarrassment^14^.

Majority of studies on empathy mentioned above are focused on how people empathize with other humans. However, recently with the increasing use of robots and virtual platforms in different sectors of our lives, chances of interacting with robots, AI, and non-human agents have increased considerably and can be expected to keep increasing. Therefore, it is necessary to build good relationships with robots as well in addition to humans^15–20^. However, are we capable of empathizing with non-human agents such as robots and virtual characters during interactions or while observing them interact with others? Humans socially interact and communicate with computers and virtual characters^21–23^. The media equation phenomenon contributes to such communication with computers and robots^24,25^. Previous studies have provided physiological evidence of humans’ ability to empathize with pain experienced by robots^16,26^. Pütten, et al. (2014) found a similar activation in the limbic system of participants during the observation of violent human–robot interactions as well as violent human–human interactions, but the neural activation was higher while observing the human–human interactions than the human–robot interactions^26^. Suzuki et al. (2015) measured electroencephalography of participants while watching humans and robots in painful situations (such as the hand being cut by a knife) and showed that while participants empathized with humanoid robots in late top-down processing in a similar manner to empathizing with humans, the beginning of the top-down process of empathy was weaker for robots compared to humans^16^. In another study that measured how people empathize with robots experiencing mistreatment by humans, participants reported a higher level of empathy with more human-looking robots and less with mechanical looking robots^27^. These studies suggest that during painful scenarios or mistreatment, people do show a tendency of showing empathy for robots as well even though it is lower compared to the level of empathy felt with other humans. Since these previous studies on empathy for robots are focused on mistreatment or pain, in this study we focused on another emotion, “empathic embarrassment” felt with robots compared to humans.

Embarrassment is an essential emotion of humans in society^28,29^, but one of the least studied emotions when it comes to previous studies on empathy. Embarrassment is a member of the family of “self-conscious” emotions that are evoked by self-reflection and self-evaluation^30^. This emotion is evoked by a wide range of apparently dissimilar situations such as being introduced to an unfamiliar audience, arriving at a social occasion under-dressed, making mistakes in front of an audience, and so on. It indeed is an unpleasant experience and causes an emotional response that has important personal and social consequences, for those observing as well as for the victim experiencing the embarrassing situation himself or herself^14^. Although we tend to consider embarrassment as an unpleasant emotion, previous studies have highlighted some social benefits of embarrassment. Child developmental studies show that embarrassment is related to social development of self^31^ and self-recognition^32^, suggesting that embarrassment is a higher-order cognitive ability. Eller et al. (2011) showed that people are more likely to be embarrassed in front of members of their own social group compared to outside social groups suggesting that embarrassment is tied to intergroup relations of people and that expressing embarrassed emotions tend to repair social relations and elicit for forgiveness^33^. Furthermore, a study by Feinberg et al. (2012) has shown that individuals who tend to express more outward signs of embarrassment also showed a tendency to be more prosocial and generous^34^. Individuals who revealed signs embarrassment were also perceived to be more trustworthy by others and the observers were willing to give resources and express a desire to affiliate with them more^34^. These studies highlight the close relationship between embarrassment and empathy.

However, only a few studies regarding empathic embarrassment (feeling embarrassed for others even though one’s own social identity is not threatened) exist so far. Miller (1987) experimentally showed that participants experienced embarrassment while another person performed a series of remarkably atypical behaviors in public such as singing, dancing, throwing a tantrum etc. at the request of the experimenter^35^. The results portrayed social embarrassment as a robust, pervasive phenomenon that nevertheless affects some people more than others^35^. Embarrassment is sometimes accompanied by distressing symptoms such as blushing, sweating, fumbling, and stuttering leading to changes in physiology of those experiencing it. Miller used skin conductance response of participants while observing the actors engaging in the embarrassing behavior as a method of measuring empathic embarrassment. We adapted this method in our study while also collecting participants’ subjective ratings to quantitively analyze empathic embarrassment and cognitive empathy. Stocks et al. (2011) revealed that when a socially desirable person is involved in an embarrassing situation, the degree of empathic embarrassment increases^14^. Thus, humans do not exhibit the same degree of empathic embarrassment for everyone. With the increasing use of robots in physical spaces and social interactions with avatars in virtual environments nowadays, it is important to know whether humans can empathize with robots or virtual avatars in embarrassing situations. Moreover, empathic embarrassment requires observers to infer whether the target agent is aware of witnesses and evaluate itself. Thus, if humans feel empathic embarrassment to robots, it suggests that humans assume the robots can be aware of being witnessed and have higher cognitive abilities such as self-reflection and self-evaluation. Therefore, studying empathic embarrassment for robots contributes to the advancement of scientific research on human perception of robots.

We utilized Virtual Reality (VR) to simulate a virtual environment in which participants were immersed with a robot character or a human character going through embarrassing situations among a crowd of people (virtual human avatars). As a control condition, we also created non-embarrassing scenes for the same situations and compared with the subjective ratings and skin conductance responses collected in the embarrassing condition. In VR literature, the capability of the VR system to elicit realistic responses is associated with the so-called Place Illusion (sense of being here despite knowing for sure one is not) and Plausibility Illusion (sense of things happening to the user despite knowing for sure they are not)^36,37^. The Plausibility Illusion is often affected by virtual characters and whether they are perceived realistic^36–38^. The failure to elicit these illusions would diminish realistic responses in users (such as feeling embarrassment towards virtual characters in our study). Therefore, in Experiment 2, we checked the plausibility of the stimuli of our Experiment 1. Both experiments were exploratory since we made no hypotheses.

## Materials and methods

### Experiment 1

#### Participants

Twenty-four participants (22 males and 2 females, mean age 22.291, SD 2.911) participated in the experiment. All participants had normal or corrected-to-normal vision. Informed consent was obtained from all the participants prior to the experiment. The experimental methods were approved by the Ethical Committee for Human-Subject Research at the Toyohashi University of Technology, and all methods were performed in accordance with the relevant guidelines and regulations. The sample size was determined via a priori power analysis using G*Power 3.1^39,40^ with a medium effect size f = 0.25, α = 0.05, power (1-β) = 0.8, repeated measures ANOVA (two avatar conditions × two embarrassment conditions).

#### Apparatus

Visual stimuli were presented using a head-mounted display (HMD; HTC VIVE Pro Eye, 1440 [width] × 1600 [height] pixels, refresh rate, 90 Hz) while the participants were seated on a chair. The stimuli were created using Unity 2019.3.0a3 on a computer (Intel Corei5 6400, NVIDIA GeForce GTX 1080 8 GB). BIOPAC MP 160 and a wireless PPG and EDA amplifier (BN-PPGED) was placed on the participants’ wrist. Two disposable electrodes (EL507) were pasted on to the distal phalanges of the participants’ index and middle fingers.

#### Stimuli and conditions

##### Visual stimuli

**Figure 1:**
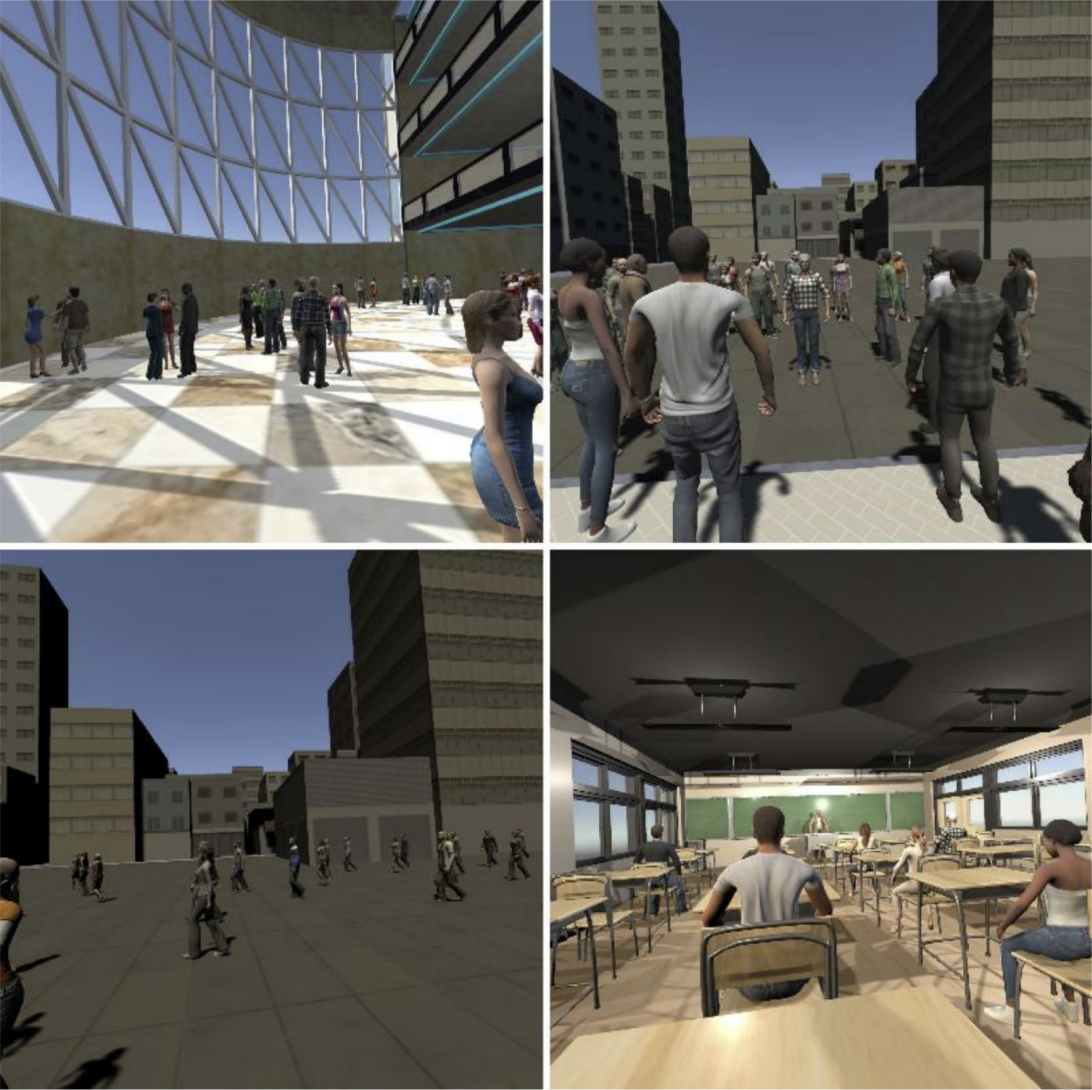
Participant’s point of view before an embarrassing or non-embarrassing scene in each situation (top-left: passing through a door, bottom-left: walking in a crowded place, top-right: dance, bottom-right: classroom). The figures were created using unity2019.3.0a3 (https://unity3d.com/get-unity/download/archive).

Two types of main actor avatars, a human avatar and a robot avatar were used for the participants to observe in 4 types of situations, walking in a crowded place, passing through an automatic door, a scenario in a classroom, and a dancing scene (Fig. 1). In each situation, the actor went through an embarrassing scenario as well as a non-embarrassing scenario. Table 1 summarizes the content in each scenario.

**Table 1:** Embarrassing and non-embarrassing situations.

Situation	Embarrassing situation	Non-embarrassing situation
Passing through a door	Bump into the door before it opens	Wait for the door to open and pass through
Walking in a crowded place	Stumble	Pick something up
Classroom	Doze off	Raise hand
Dance	Dance unskillfully in front of many people	Dance unskillfully in an empty space

##### Conditions

**Figure 2:**
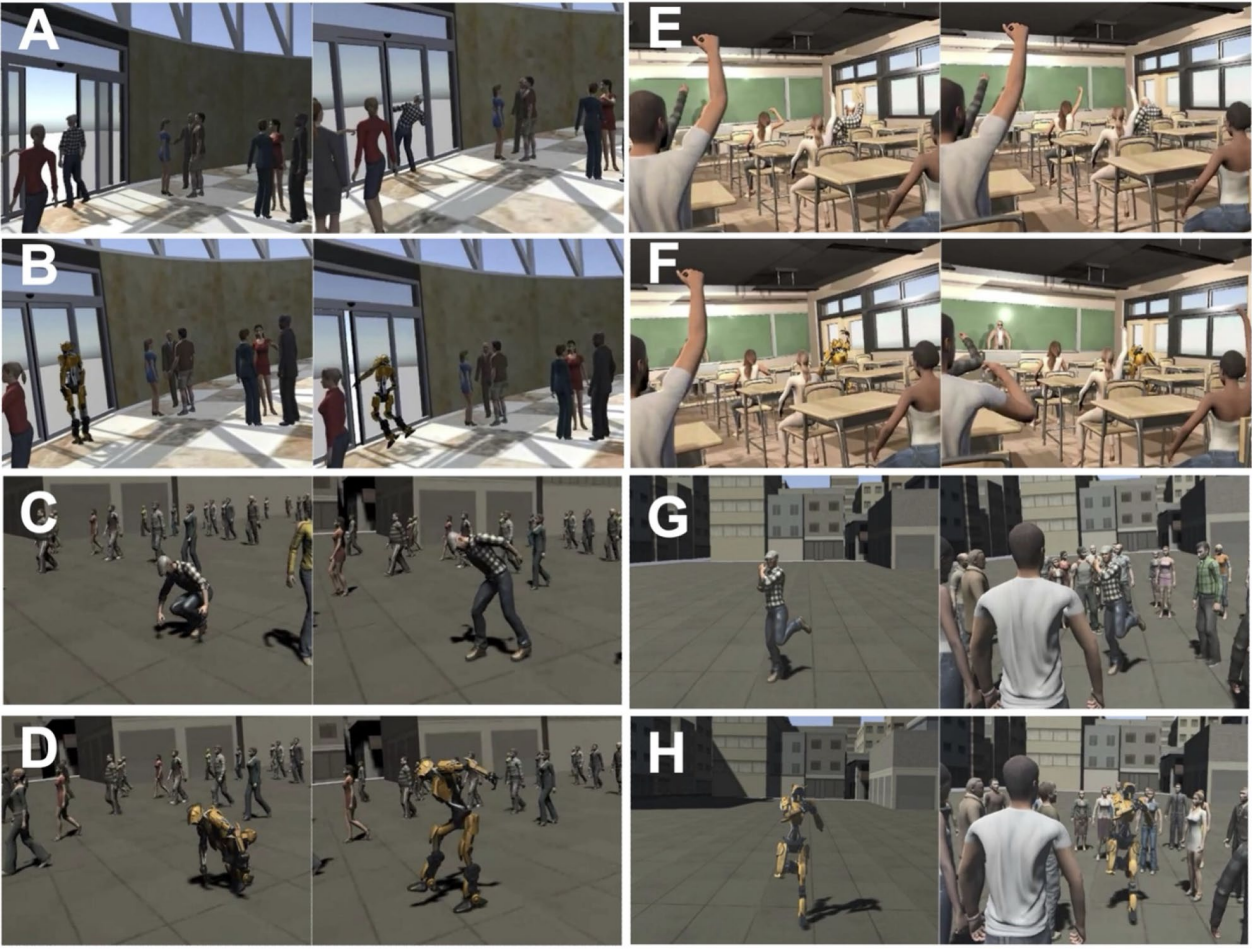
(**A**) Human avatar passing through a door (left: non-embarrassing scenario, right: embarrassing scenario). (**B**) Robot avatar passing through a door (left: non-embarrassing scenario, right: embarrassing scenario). (**C**) Human walking in a crowded place (left: non-embarrassing scenario, right: embarrassing scenario). (**D**) Robot walking in a crowded place (left: non-embarrassing scenario, right: embarrassing scenario). (**E**) Human in classroom (left: non-embarrassing scenario, right: embarrassing scenario). (**F**) Robot in classroom (left: non-embarrassing scenario, right: embarrassing scenario). (**G**) Human dancing (left: non-embarrassing scenario, right: embarrassing scenario). (**H**) Robot dancing (non-embarrassing scenario, right: embarrassing scenario). The figures were created using unity2019.3.0a3 (https://unity3d.com/get-unity/download/archive).

There were 16 types of stimuli (avatar type of actor: human/robot x Embarrassment: embarrassed/non-embarrassed x situation: passing through a door, walking in a crowded place, classroom, dance) in the experiment as shown in Fig. 2 and Supplementary video 1. However, the scores/skin conductance of the 4 situations were averaged into one for each combination of avatar type and embarrassment condition, giving a total of 4 conditions. In each situation, many people (avatars) were placed around the actor.

##### Questionnaire

Participants rated their self-embarrassment (the participant’s own feeling) and actor-embarrassment (actor’s feeling guessed or perceived by the participant) at the end of each trial using the touch pad and the trigger button of the controller. The answers were recorded in a 7-point Likert scale where the scores from -3 to 3 corresponded to the answers shown below in Table 2.

**Table 2:** Answers for self or actor embarrassment and corresponding scores.

Score in Likert scale	Feeling
-3	Extremely embarrassing
-2	Embarrassing
-1	Slightly embarrassing
0	Neither embarrassing nor proud
1	Slightly proud
2	Proud
3	Extremely proud

A 7-pont Likert scale was chosen over a 5-point Likert scale since 7-point scales provide a more accurate measure of a participant's true evaluation and are more appropriate for assessing usability according to some previous studies^41,42^. Furthermore, the fact that only two questionnaire items were asked in our experiment gives participants enough time to discreetly choose an answer from a 7-point scale.

#### Procedure

The subjects were presented with 16 scenarios (2 avatar types × 2 embarrassment conditions × 4 situations), one at a time in a random order while they were seated immersed in the virtual environment corresponding to each situation. At the beginning of each trial, the participants were asked to look around and spot the actor and focus on the actor till the end of the trial. Fifteen to seventeen seconds into each trial, an embarrassing or a non-embarrassing event occurred to the actor. Each session lasted thirty to forty seconds, and skin conductance was measured throughout the session. At the end of the trial, participants answered a questionnaire regarding how they felt in the trial. Additionally, during each trial, the skin conductance levels of participants were measured and recorded using a wireless transmitter attached to the wrist of the participant that was connected to two electrodes on the fingers.

### Experiment 2

#### Participants

Twenty-four participants (all males, mean age 23.875, SD 3.261) participated in the experiment. All participants had normal or corrected-to-normal vision. Informed consent was obtained from all the participants prior to the experiment. The experimental methods were approved by the Ethical Committee for Human-Subject Research at the Toyohashi University of Technology, and all methods were performed in accordance with the relevant guidelines and regulations. Similar to Experiment 1, the sample size was determined via a priori power analysis using G*Power 3.1^39,40^ with a medium effect size f = 0.25, α = 0.05, power (1-β) = 0.8, repeated measures ANOVA (two avatar conditions × two embarrassment conditions).

#### Apparatus

Apparatus was the same as in Experiment 1. However, Skin Conductance was not measured in Experiment 2.

#### Stimuli and conditions

##### Visual stimuli

The same visual stimuli used in Experiment 1 were used in randomized order for each participant.

##### Conditions

The conditions were the same as those in Experiment 1.

##### Questionnaire

Participants answered the questions shown in Table 3 at the end of each trial in a 7-point Likert scale ranging from -3 (Not at all) to + 3 (Totally). The question order was randomized each time for each participant. The questionnaire items were adapted from a recent previous study that measured plausibility of virtual events of an experiment conducted in VR^38^.

**Table 3:** Questions relating to plausibility, that the events observed in VR were really happening. All scores are on a -3 (Not at all) to + 3 (Totally) scale.

Variable name	Question
*real*	To what extent did you behave, and respond as if the situation were real?
*emotion*	To what extent was your emotional response the same as if the situation had been real?
*thoughts*	To what extent were the thoughts you had the same as if the situation had been real?
*auto real*	To what extent did you find yourself surprisingly behaving as if the situation were real even though you knew it was not real?
*actor real*	To what extent did you behave, and respond as if the main actor were a real person/robot?
*emotion to actor*	To what extent were your emotional responses to the main actor the same as if he/it were real?
*thoughts to actor*	To what extent were your thoughts in relation to the main actor the same as if he/it were real?

## Procedure

The subjects were presented the same 16 scenarios from Experiment 1 (2 avatar types × 2 embarrassment conditions × 4 situations), one at a time in a random order while they were seated immersed in the virtual environment corresponding to each situation. At the beginning of each trial, the participants were asked to look around and spot the actor and focus on the actor till the end of the trial. Fifteen to seventeen seconds into each trial, an embarrassing or a non-embarrassing event occurred to the actor. At the end of the trial, participants answered a questionnaire regarding plausibility.

### Ethics statement

The experimental methods were approved by the Ethical Committee for Human-Subject Research at Toyohashi University of Technology, and all methods were strictly performed in accordance with relevant guidelines and regulations.

## Results

### Experiment 1

#### Self-embarrassment (empathic embarrassment)

**Figure 3:**
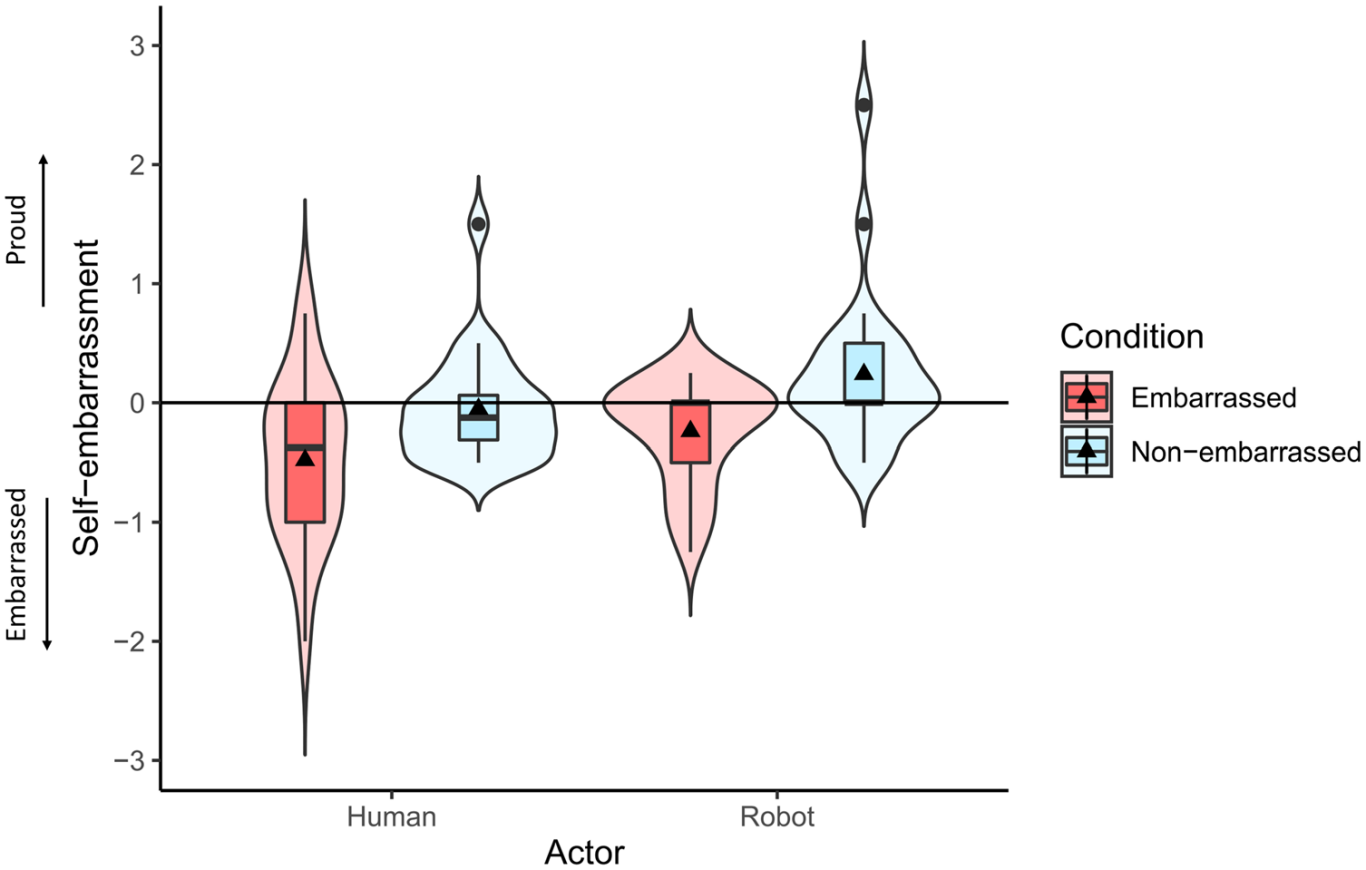
Results of subjective scores of self-embarrassment.

We calculated the self-embarrassment (see Fig. 3) towards the human and robot actors in embarrassed and non-embarrassed conditions. A two-way repeated measures ANOVA with ART (aligned rank transformation) procedure^43^ was conducted since some of the data significantly deviated from normality according to the results of Shapiro–Wilk tests (embarrassed condition for human avatar: W = 0.967, *p* = 0.594, non-embarrassed condition for human avatar: W = 0.819 , p < 0.001, embarrassed condition for robot avatar: W = 0.868, *p* = 0.005, non-embarrassed condition for robot avatar: W = 0.792, p < 0.001).

No significant interaction was observed between the avatar type and embarrassment condition (F(1,23) = 0.079, *p* = 0.781, ${\eta }_{p}^{2}$=0.003). However, significant main effects were observed for the avatar type (F(1,23) = 8.279, *p* = 0.009, ${\eta }_{p}^{2}$=0.265) as well as for the embarrassment condition (F(1,23) = 11.248, *p* = 0.003, ${\eta }_{p}^{2}$=0.328). These indicated that the self-embarrassment was significantly higher (higher negative score) towards the human avatar compared to the robot avatar in both embarrassed and non-embarrassed conditions, and significantly higher in the embarrassed condition compared to the non-embarrassed condition for both human actor and robot actor.

#### Actor-embarrassment (cognitive empathy)

**Figure 4:**
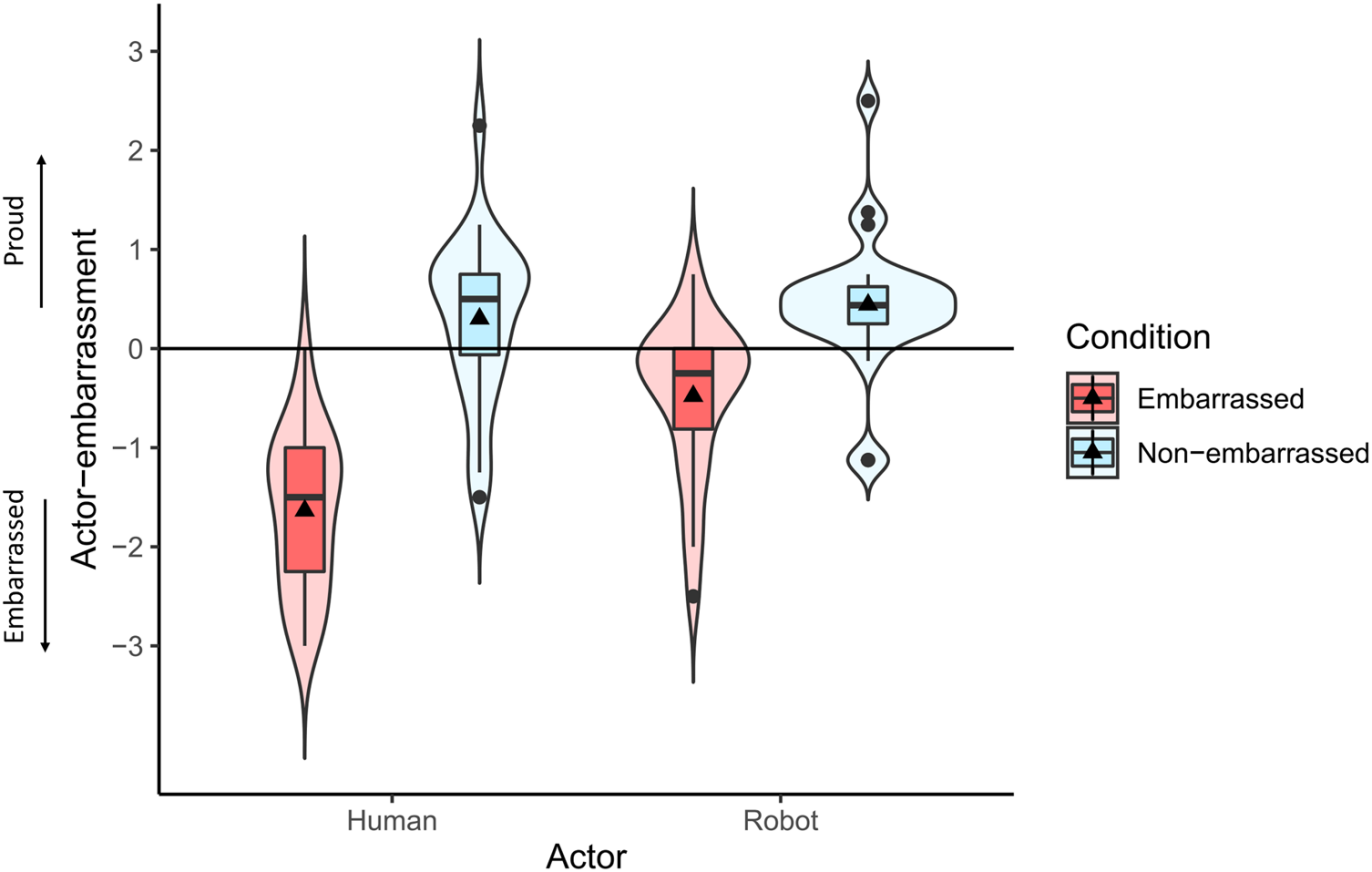
Results of subjective scores of actor-embarrassment.

Next, we calculated actor-embarrassment (see Fig. 4) towards the human and robot actors in embarrassed and non-embarrassed conditions. A two-way repeated measures ANOVA with ART (aligned rank transformation) procedure^43^ was conducted since some of the data significantly deviated from normality according to the results of Shapiro–Wilk tests (embarrassed condition for human avatar: W = 0.963 *p* = 0.496 , non-embarrassed condition for human avatar: W = 0.947, *p* = 0.233, embarrassed condition for robot avatar: W = 0.902, *p* = 0.024, non-embarrassed condition for robot avatar: W = 0.867, *p* = 0.005).

A significant interaction was observed between the avatar type and embarrassment condition (F(1,23) = 33.447, p < 0.001, ${\eta }_{p}^{2}$=0.593). Simple main effects analysis showed that the actor-embarrassment was significantly higher (higher negative score) towards the human actor than towards the robot actor in the embarrassed condition (F(1,23) = 54.135, p < 0.001, ${\eta }_{p}^{2}$=0.702). Furthermore, for both human and robot actors, actor-embarrassment was significantly higher (higher negative value) in the embarrassed condition compared to the non-embarrassed condition (human actor: F(1,23) = 101.14, p < 0.001, ${\eta }_{p}^{2}$=0.815, robot actor: F(1,23) = 71.196, p < 0.001, ${\eta }_{p}^{2}$=0.756).

#### Skin conductance response (SCR)

**Figure 5:**
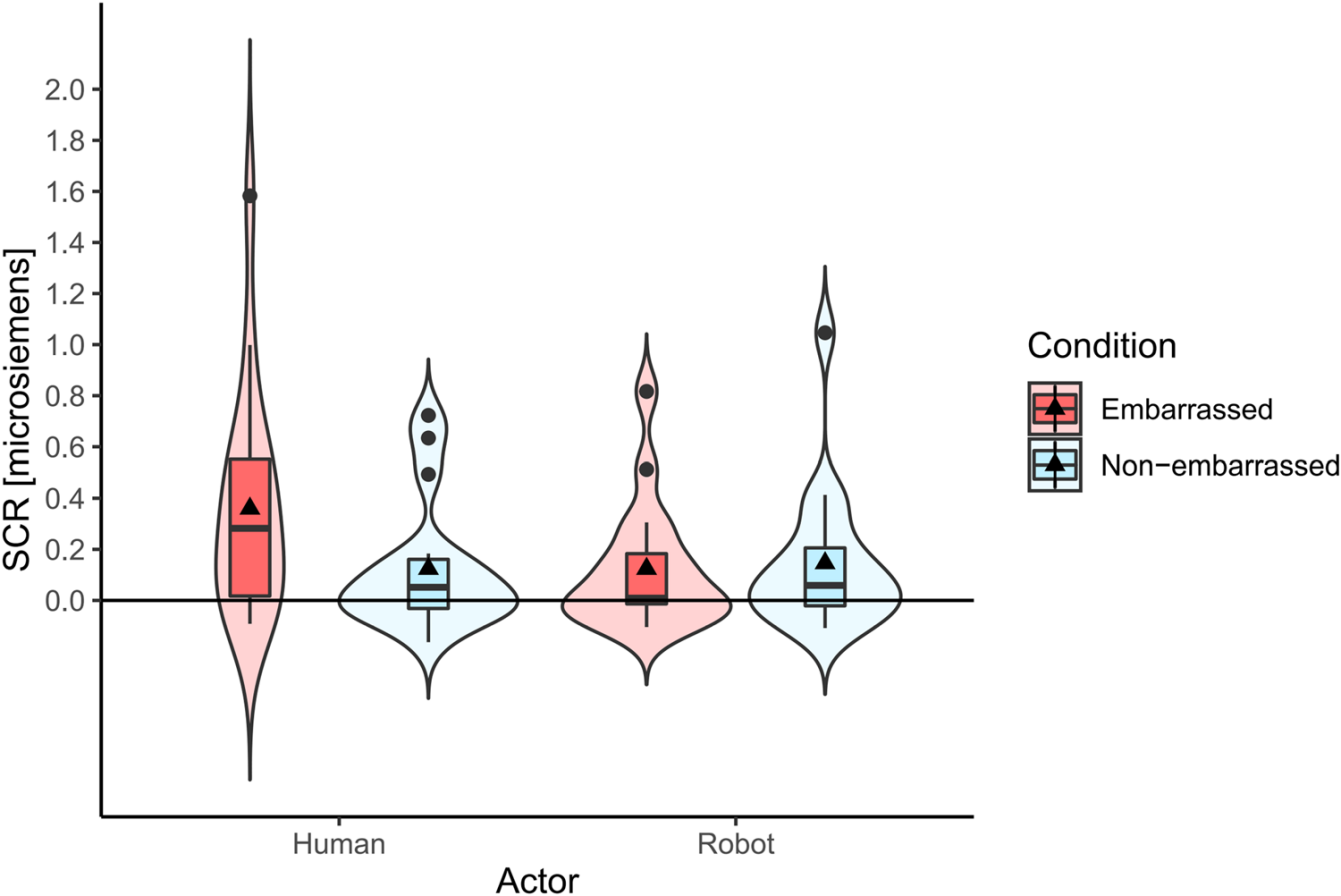
Skin conductance response. Maximum skin conductance detected in the first five seconds after the embarrassing event compared to the baseline calculated before the embarrassing event.

Skin conductance response of 17 participants were analyzed, removing the data of 7 participants due to high noise ratios or technical errors in reading the data during the experiment. The average of skin conductance read in 3 s just before the embarrassing or the non-embarrassing event occurred was first calculated as the baseline. For each condition, the difference between the baseline and the maximum skin conductance read in the first 5 s since the beginning of the embarrassing/non-embarrassing event was calculated and averaged. Figure 5 shows the summary of the calculated results in microsiemens.

A two-way repeated measures ANOVA was conducted and a marginally significant interaction was observed between the avatar type and embarrassment condition (F(1,16) = 3.088, *p* = 0.098, ${\eta }_{p}^{2}$=0.162). Simple main effects analysis showed that the skin conductance response was marginally significantly higher in the embarrassed condition compared to the non-embarrassed condition for the human avatar (F(1,16) = 3.949, *p* = 0.0643, ${\eta }_{p}^{2}$=0.198). Furthermore, compared to the robot avatar, the skin conductance response towards the human avatar in the embarrassed condition was also marginally significantly higher (F(1,16) = 4.299, *p* = 0.055, ${\eta }_{p}^{2}$=0.212).

### Experiment 2

#### Results of the plausibility questionnaire

**Figure 6:**
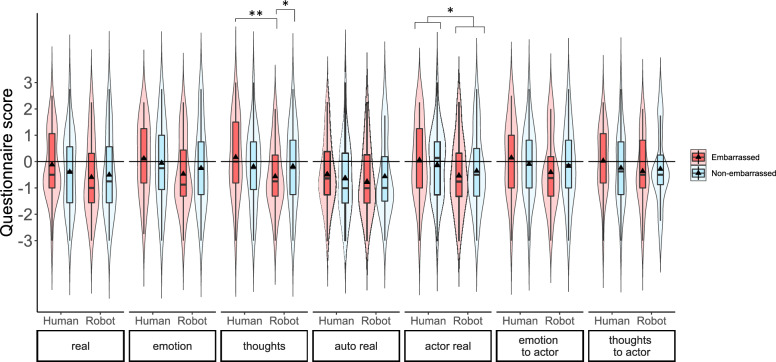
Results of the plausibility questionnaire.

Figure 6 shows the results for each questionnaire item in Table 3. All data gathered with the plausibility questionnaire were confirmed to be normally distributed according to Shapiro–Wilk tests (*p* = 0.995 ~ 0.114). Therefore, two-way repeated measures ANOVAs were conducted for each questionnaire item.

First, for the *real* item of the questionnaire, a two-way repeated measures ANOVA was conducted. No main effects or interactions were found (Avatar type: F(1,23) = 3.186, *p* = 0.088, ${\upeta }_{\mathrm{p}}^{2}$=0.122; Embarrassment condition: F(1,23) = 0.579, *p* = 0.455, ${\upeta }_{\mathrm{p}}^{2}$=0.025; Interaction: F(1,23) = 3.412, *p* = 0.078, ${\upeta }_{\mathrm{p}}^{2}$=0.129).

Next, a two-way repeated measures ANOVA was conducted for the *emotion* item of the questionnaire. No main effects or interactions were found (Avatar type: F(1,23) = 3.893, *p* = 0.061, ${\upeta }_{\mathrm{p}}^{2}$=0.145; Embarrassment condition: F(1,23) = 0.038, *p* = 0.847, ${\upeta }_{\mathrm{p}}^{2}$=0.002; Interaction: F(1,23) = 1.982, *p* = 0.173, ${\upeta }_{\mathrm{p}}^{2}$=0.079).

For the *thoughts* item of the questionnaire, a two-way repeated measures ANOVA showed a significant interaction between the avatar type and the embarrassment condition (F(1,23) = 7.755, *p* = 0.011, ${\eta }_{p}^{2}$=0.252). Simple main effects analysis showed that in the embarrassed condition, the score for the human actor was significantly higher than the score for the robot actor (F(1,23) = 10.439, *p* = 0.004, ${\eta }_{p}^{2}$=0.312). Furthermore, for the robot actor, the score in the non-embarrassed condition was significantly higher than the score in the embarrassed condition (F(1,23) = 5.806, *p* = 0.024, ${\eta }_{p}^{2}$=0.202). Main effects were not significant (Avatar type: F(1,23) = 3.794, *p* = 0.064, ${\upeta }_{\mathrm{p}}^{2}$=0.142; Embarrassment condition: F(1,23) = 0.011, *p* = 0.912, ${\upeta }_{\mathrm{p}}^{2}$=0.001).

For the *auto real* item of the questionnaire, two-way repeated measures ANOVA showed no significant interaction (F(1,23) = 1.355, *p* = 0.257, ${\eta }_{p}^{2}$=0.056) or main effects (avatar type: F(1,23) = 0.388, *p* = 0.540, ${\eta }_{p}^{2}$=0.017, embarrassment condition: F(1,23) = 0.022, *p* = 0.883, ${\eta }_{p}^{2}$=0.001).

For the *actor real* item of the questionnaire, a significant main effect on avatar type was observed with the score for the human actor being significantly higher than the score for the robot actor (F(1,23) = 4.626, *p* = 0.042, ${\eta }_{p}^{2}$=0.167). No significant main effect on embarrassment condition (F(1,23) = 0.001, *p* = 0.970, ${\eta }_{p}^{2}$=0.000), or significant interaction between was observed (F(1,23) = 2.135, *p* = 0.158, ${\eta }_{p}^{2}$=0.085).

For the *emotion to actor* item of the questionnaire, a significant interaction between the avatar type and the embarrassment condition was observed (F(1,23) = 4.548, *p* = 0.044, ${\eta }_{p}^{2}$=0.165). Simple main effects analysis did not show any significant difference, but in the embarrassed condition, the score for the human actor was marginally higher than the score for the robot actor (F(1,23) = 3.947, *p* = 0.059, ${\eta }_{p}^{2}$=0.147).

For the *thoughts to actor* item of the questionnaire, two-way repeated measures ANOVA did not show a significant interaction (F(1,23) = 2.589, *p* = 0.121, ${\eta }_{p}^{2}$=0.101) or main effects (avatar type: F(1,23) = 1.356, *p* = 0.256, ${\eta }_{p}^{2}$=0.056, embarrassment condition: F(1,23) = 0.460, *p* = 0.504, ${\eta }_{p}^{2}$=0.019).

#### Overall plausibility (average of all questions of the plausibility questionnaire)

**Figure 7:**
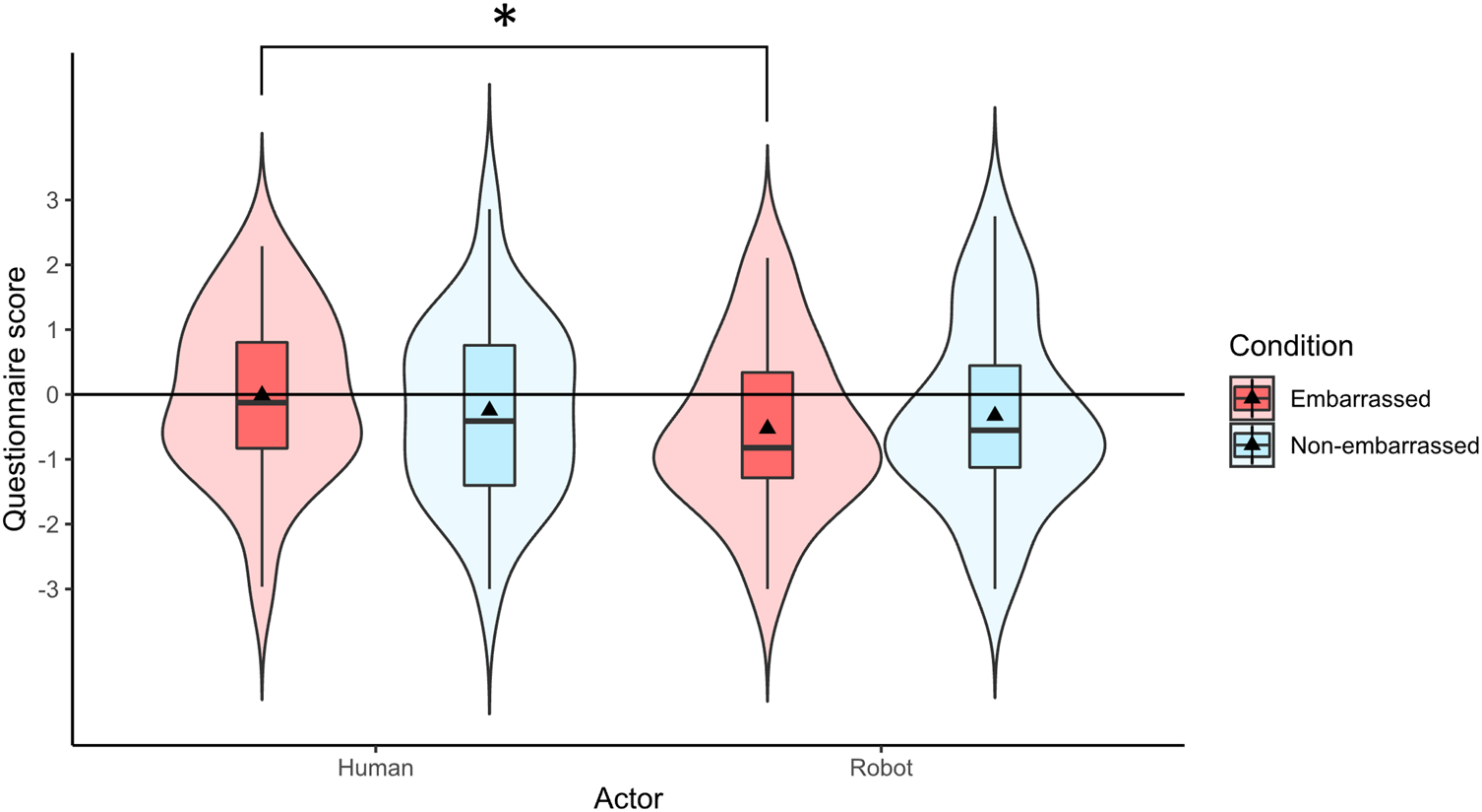
Averaged scores of all questions in the plausibility questionnaire.

Cronbach’s alpha of the plausibility questionnaire was 0.983. Thus, we calculated the average of all items (Fig. 7). A two-way repeated measures ANOVA was conducted since the data did not deviate from normality according to the results of Shapiro–Wilk tests (embarrassed condition for human avatar: W = 0.981, *p* = 0.909, non-embarrassed condition for human avatar: W = 0.980, *p* = 0.890, embarrassed condition for robot avatar: W = 0.976, *p* = 0.820, non-embarrassed condition for robot avatar: W = 0.971, *p* = 0.698). A significant interaction between the avatar type and embarrassment condition were observed (F(1,23) = 4.716, *p* = 0.040, ${\eta }_{p}^{2}$=0.170). Simple main effects analysis showed that in the embarrassed condition, the score for the human actor was significantly higher than the score for the robot actor (F(1,23) = 6.712, *p* = 0.016, ${\eta }_{p}^{2}$=0.226).

## Discussion

### Summary of results

In Experiment 1, we investigated how humans feel empathic embarrassment and cognitive empathy (related to embarrassment) with robots compared to humans in four different virtual environments. Self-embarrassment showed no interaction between the actor type and embarrassment condition but showed significant main effects for both actor type and embarrassment condition with self-embarrassment being higher with the human actor than with the robot actor and higher in the embarrassed condition compared to the non-embarrassed condition. Actor-embarrassment showed a significant interaction between the actor type and embarrassment condition with significant simple main effects showing that the scores in the embarrassed condition were higher than the scores in the non-embarrassed condition for each actor (human and robot) and the score for the embarrassed condition was significantly higher with the human actor compared to that with the robot actor. Skin conductance showed a higher (but not significant) response with the human actor compared to that with the robot actor in the embarrassed condition.

In Experiment 2, we investigated the plausibility of the stimuli used in Experiment 1. The participants thought the situation was significantly more real in the embarrassed condition with the human avatar compared to the embarrassed condition with the robot avatar (*thoughts* item). They also thought the situation was more real in the non-embarrassed condition with the robot avatar compared to the embarrassed condition with the robot avatar (*thoughts* item). Furthermore, the human avatar was perceived to be more real compared to the robot avatar regardless of the embarrassed or non-embarrassed events that occurred in the scene (*actor real* item). The average scores of all items in the plausibility questionnaire also showed a significant difference between the embarrassed condition with the human avatar compared to the embarrassed condition with the robot avatar. Other items in the questionnaire (*real, emotion, auto real, emotion to actor, and thoughts to actor* items) did not show significant differences between experiment conditions.

### Significant empathic embarrassment for both human and robot actors

Regardless of the avatar type of the actor, self-embarrassment and actor-embarrassment were both significantly higher when the actor faced an embarrassing incident (embarrassed condition) compared to when no embarrassing incident happened (non-embarrassed condition). This suggests that the situations chosen in this experiment in general were indeed considered as embarrassing events by the participants and the embarrassment was significantly higher than that in the non-embarrassed condition towards the robot actor as well as the human actor. Cronbach’s alpha of two questionnaire items (self-embarrassment and acter-embarrassment) was 0.615. Thus, these two items have some consistency, but reflect different aspects of empathic embarrassment and cognitive empathy, respectively. The empathic embarrassment was not only cognitive inference, but also felt as self-embarrassment. Thus, humans can empathize with robots in embarrassing situations, suggesting that humans assume the robots can be aware of being witnessed and have some degree of self-consciousness based on self-reflection and self-evaluation.

However, the appearance of the robot may affect the empathic embarrassment because humans empathize more strongly with more human-looking robots and less with more mechanical-looking robots when they are mistreated by humans^27^. Subjective ratings of empathic embarrassment for the human and the robot were not significantly different. It may be because we used a humanoid robot that had two arms, two legs, a head, and similar joints and proportions to humans. We should investigate the effect of robot appearance on empathic embarrassment in the future.

### Lower actor-embarrassment with the robot actor compared to the human actor.

When we consider only the embarrassed condition, the inferred actor-embarrassment was significantly higher with the human avatar compared to that with the robot avatar. This suggests that cognitive empathy with regards to embarrassment is lower for robots compared to humans and this result is similar to previous studies that investigated empathy for robots during mistreatment^27^ and pain^16^. It may be due to naive knowledge that robots do not have a mind.

Humans are less concerned about abusing robots compared to abusing other humans^25^ and children sometimes abuse social robots in public spaces^44^. The low cognitive empathy to robot embarrassment as well as the low cognitive empathy to robot pain^16^ may be related to these phenomena.

### Skin conductance response

Skin conductance showed a higher (marginally significant) value towards the human actor compared to the robot actor in the embarrassed condition. Therefore overall, these results suggest that while we feel a certain level of empathic embarrassment and cognitive empathy towards robots, it is significantly less than that felt towards humans. However, the difference was not statistically significant. It may be caused by underpower. The data of 7 participants were removed out of 24 participants due to high noise ratios or technical errors during the experiment.

### Plausibility of the experiment stimuli.

The average plausibility in the embarrassed condition was significantly higher with the human avatar compared to the robot avatar. However, *real, emotion, auto real, emotion to actor, and thoughts to actor* items (see Fig. 6) were not significantly different among the experiment conditions. Therefore, part of the difference between human and robot avatars might be due to the difference of their plausibility, but it is not supposed that the empathic embarrassment, which is an emotion, was affected solely by the difference in plausibility of conditions.

### Limitations

The study was limited to virtual environments and the actors were 3D avatars. Therefore, there is a possibility of participants feeling less empathy or emotion for them compared to real humans or physical robots. Furthermore, skin conductance responses showed only marginally significant results this time. The data loss of seven participants resulting a sample size of 17 for skin conductance data also may reduce the reliability of the results, which is a limitation of the current study. Furthermore, other measurements such as electroencephalography may provide better insights for physiological changes with regards to empathic emotions.

We measured embarrassment and pride in one scale in this experiment since embarrassment, and pride are both known to be members of a family of “self-conscious” emotions^30^ and we assumed pride to be the opposite emotion of embarrassment. Thus, we adopted a Likert scale from -3 (Extremely embarrassing) and + 3 (Extremely proud), while the previous studies used the scale from 1 (not at all) to 7 (very much embarrassing). In a pilot study, participants felt our scale was better than the previous one to evaluate the movie scenes. However, the relationship between embarrassment and pride should be further studied in future studies.

It is known that embarrassment is affected by cultures^45,46^. Familiarity and attitude to robots are affected by cultures as well^47,48^. However, participants of our study were mostly Japanese. Cross-cultural investigation is needed in future studies.

Dancing unskillfully in front of a crowd was considered embarrassing in this experiment compared to performing the same dance alone. However, one may argue that dancing in front of a crowd may instead show confidence and be perceived as a proud act instead of an embarrassing act. To check if the participants really found the dance scenes embarrassing or not, we analyzed the subjective ratings for self and actor-embarrassment only for the dance scenes of Experiment 1. Two-way repeated measures ANOVA with aligned rank transform (due to non-parametric data) showed that there was a significant main effect on the avatar type with the ratings for both self and actor-embarrassment for the human avatar showing a significantly higher embarrassment compared to the ratings for the robot avatar (Self-embarrassment: F(1,23) = 8.832, *p* = 0.007, ${\eta }_{p}^{2}$=0.277, Actor-embarrassment: F(1,23) = 7.769, *p* = 0.010, ${\eta }_{p}^{2}$=0.253). However, there was no significant main effect on Condition (embarrassing/non-embarrassing) and there was no significant interaction between the condition and avatar type for both self-embarrassment and actor-embarrassment. Therefore, choosing stimuli that differentiate embarrassing and non-embarrassing events better might be suited for future experiments related to embarrassment.

The current study was not diverse with regards to gender due to the majority of male participants. Previous studies have shown an apparent gender effect associated with empathy, with a clear female superiority in empathic capability^49^. Therefore, further studies taking gender into account may be necessary to further understand the ability of humans to empathize with robots.

The overall score for the *actor real* item of the plausibility questionnaire was significantly higher towards the human avatar compared to the robot avatar, meaning that the human avatar was perceived more like a real human compared to the robot avatar being perceived as a real robot. This difference in actor realism could be due to the fact that we are not used to watching robots in real life as frequently as we see other humans. The difference in animations used for human, and robot avatars may also be another reason for this plausibility difference, and it is possible that the embarrassment scores were affected by this.

## Conclusions

We examined whether humans feel empathic embarrassment with robots in virtual environments. Both empathic embarrassment and cognitive empathy were rated significantly higher in the embarrassing situations compared to the non-embarrassed situations with both human and robot actors, and the cognitive empathy was higher with the human actor compared to the robot actor. These results suggest that while we feel a certain level of empathic embarrassment and cognitive empathy with regards to embarrassment with robots, it is significantly less than that felt with humans. The difference in the plausibility of experiment stimuli also may be a possible reason for the difference in empathic embarrassment between the human and robot avatars.

### Supplementary Information

Supplementary Information 1.

Supplementary Video 1.

## Data Availability

The datasets obtained for each participant and analyzed during the current study are available from the corresponding author on reasonable request.

## References

[1] Eisenberg, N., & Miller, P. A. (1990). 13 Empathy, sympathy, and altruism: Empirical and conceptual links. *Empathy Dev*, 292.

[2] Davis MH, Luce C, Kraus SJ (1994). The heritability of characteristics associated with dispositional empathy. J. Pers..

[3] Batson CD, Batson JG, Slingsby JK, Harrell KL, Peekna HM, Todd RM (1991). Empathic joy and the empathy-altruism hypothesis. J. Pers. Soc. Psychol..

[4] Batson CD, Early S, Salvarani G (1997). Perspective taking: Imagining how another feels versus imaging how you would feel. Pers. Soc. Psychol. Bull..

[5] Smith KD, Keating JP, Stotland E (1989). Altruism reconsidered: The effect of denying feedback on a victim’s status to empathic witnesses. J. Pers. Soc. Psychol..

[6] Hoffman, M. L. (2001). Toward a comprehensive empathy-based theory of prosocial moral development.

[7] Krebs D (1975). Empathy and altruism. J. Pers. Soc. Psychol..

[8] Jackson PL, Meltzoff AN, Decety J (2005). How do we perceive the pain of others? A window into the neural processes involved in empathy. Neuroimage.

[9] Lamm C, Nusbaum HC, Meltzoff AN, Decety J (2007). What are you feeling? Using functional magnetic resonance imaging to assess the modulation of sensory and affective responses during empathy for pain. PLoS ONE.

[10] Cheng Y, Lin CP, Liu HL, Hsu YY, Lim KE, Hung D, Decety J (2007). Expertise modulates the perception of pain in others. Curr. Biol..

[11] Gu X, Han S (2007). Attention and reality constraints on the neural processes of empathy for pain. Neuroimage.

[12] Montada L, Schneider A (1989). Justice and emotional reactions to the disadvantaged. Soc. Just. Res..

[13] Vitaglione GD, Barnett MA (2003). Assessing a new dimension of empathy: Empathic anger as a predictor of helping and punishing desires. Motiv. Emot..

[14] Stocks EL, Lishner DA, Waits BL, Downum EM (2011). I’m embarrassed for you: The effect of valuing and perspective taking on empathic embarrassment and empathic concern. J. Appl. Soc. Psychol..

[15] Rosenthal-von der Pütten AM, Krämer NC, Hoffmann L, Sobieraj S, Eimler SC (2013). An experimental study on emotional reactions towards a robot. Int. J. Soc. Robot..

[16] Suzuki Y, Galli L, Ikeda A, Itakura S, Kitazaki M (2015). Measuring empathy for human and robot hand pain using electroencephalography. Sci. Rep..

[17] Broadbent E (2017). Interactions with robots: The truths we reveal about ourselves. Annu. Rev. Psychol..

[18] Wiese E, Metta G, Wykowska A (2017). Robots as intentional agents: using neuroscientific methods to make robots appear more social. Front. Psychol..

[19] Hortensius R, Hekele F, Cross ES (2018). The perception of emotion in artificial agents. IEEE Trans. Cognit. Dev. Syst..

[20] Henschel A, Hortensius R, Cross ES (2020). Social cognition in the age of human–robot interaction. Trends Neurosci..

[21] Nass C, Moon Y, Green N (1997). Are machines gender neutral? Gender-stereotypic responses to computers with voices. J. Appl. Soc. Psychol..

[22] Hoffmann, L., Krämer, N. C., Lam-chi, A. & Kopp, S. Media equation revisited: Do users show polite reactions towards an embodied agent? *Intelligent Virtual Agents Lecture Notes in Computer Science*. Ruttkay, Z., Kipp, M., Nijholt, A. & Vilhjálmsson, H. H. (eds.) 159–165 (Springer, Berlin, 2009).

[23] Von der Pütten, A. M., Krämer, N. C., Gratch, J., & Kang, S. H. (2010). “It doesn’t matter what you are!” explaining social effects of agents and avatars. *Comput. Hum. Behav*.

[24] Reeves B, Nass C (1996). The media equation: How people treat computers, television, and new media like real people. Cambridge, UK.

[25] Bartneck C, Hu J (2008). Exploring the abuse of robots. Interact. Stud..

[26] Rosenthal-Von Der Pütten AM, Schulte FP, Eimler SC, Sobieraj S, Hoffmann L, Maderwald S, Krämer NC (2014). Investigations on empathy towards humans and robots using fMRI. Comput. Hum. Behav..

[27] Riek, L. D., Rabinowitch, T. C., Chakrabarti, B., & Robinson, P. (2009, March). How anthropomorphism affects empathy toward robots. In *Proceedings of the 4th ACM/IEEE International Conference on Human Robot Interaction* (pp. 245–246).

[28] Edelmann RJ (1987). The Psychology of Embarrassment.

[29] Miller RS, Tangney JP (1994). Differentiating embarrassment and shame. J. Soc. Clin. Psychol..

[30] Tangney, J. P. (1999). The self-conscious emotions: Shame, guilt, embarrassment and pride.

[31] Buss AH, Iscoe I, Buss EH (1979). The development of embarrassment. J. Psychol..

[32] Lewis, M., Sullivan, M. W., Stanger, C., & Weiss, M. (1989). Self development and self-conscious emotions. *Child Development*, 146–156. 2702864

[33] Eller A, Koschate M, Gilson KM (2011). Embarrassment: The ingroup–outgroup audience effect in faux pas situations. Eur. J. Soc. Psychol..

[34] Feinberg M, Willer R, Keltner D (2012). Flustered and faithful: Embarrassment as a signal of prosociality. J. Pers. Soc. Psychol..

[35] Miller RS (1987). Empathic embarrassment: Situational and personal determinants of reactions to the embarrassment of another. J. Pers. Soc. Psychol..

[36] Slater M (2009). Place illusion and plausibility can lead to realistic behaviour in immersive virtual environments. Philos. Trans. R. Soc. B Biol. Sci..

[37] Slater M, Banakou D, Beacco A, Gallego J, Macia-Varela F, Oliva R (2022). A separate reality: an update on place illusion and plausibility in virtual reality. Front. Virtual Real..

[38] Neyret S, Navarro X, Beacco A, Oliva R, Bourdin P, Valenzuela J, Slater M (2020). An embodied perspective as a victim of sexual harassment in virtual reality reduces action conformity in a later milgram obedience scenario. Sci. Rep..

[39] Faul F, Erdfelder E, Lang AG, Buchner A (2007). G* Power 3: A flexible statistical power analysis program for the social, behavioral, and biomedical sciences. Behav. Res. Methods.

[40] Faul F, Erdfelder E, Buchner A, Lang AG (2009). Statistical power analyses using G* Power 3.1: Tests for correlation and regression analyses. Behav. Res. Methods.

[41] Joshi A, Kale S, Chandel S, Pal DK (2015). Likert scale: Explored and explained. Br. J. Appl. Sci. Technol..

[42] Finstad K (2010). Response interpolation and scale sensitivity: Evidence against 5-point scales. J. Usability Stud..

[43] Wobbrock, J. O., Findlater, L., Gergle, D., & Higgins, J. J. (2011). The aligned rank transform for nonparametric factorial analyses using only anova procedures. In *Proceedings of the SIGCHI Conference on Human Factors in Computing Systems *(pp. 143–146).

[44] Nomura, T., Uratani, T., Kanda, T., Matsumoto, K., Kidokoro, H., Suehiro, Y., & Yamada, S. (2015, March). Why do children abuse robots?. In *Proceedings of the tenth Annual ACM/IEEE International Conference on Human-Robot Interaction Extended abstracts *(pp. 63–64).

[45] Sueda K, Wiseman RL (1992). Embarrassment remediation in Japan and the United States. Int. J. Intercult. Relat..

[46] Imahori TT, Cupach WR (1994). A cross-cultural comparison of the interpretation and management of face: US American and Japanese responses to embarrassing predicaments. Int. J. Intercult. Relat..

[47] Haring, K. S., Silvera-Tawil, D., Matsumoto, Y., Velonaki, M., & Watanabe, K. (2014, October). Perception of an android robot in Japan and Australia: A cross-cultural comparison. In *International Conference on Social Robotics* (pp. 166–175). Springer, Cham.

[48] MacDorman KF, Green RD, Ho CC, Koch CT (2009). Too real for comfort? Uncanny responses to computer generated faces. Comput. Hum. Behav..

[49] Baron-Cohen S, Wheelwright S (2004). The empathy quotient: an investigation of adults with Asperger syndrome or high functioning autism, and normal sex differences. J. Autism Dev. Disord..

[50] Sugiura, M., Higashihata, K., Sato, A., Itakura, S., and Kitazaki, M. (2020). Empathy with human’s and robot’s embarrassments in virtual environments, ICAT-EGVE 2020. Poster.

