# Supplementary material for: Empathic embarrassment towards non-human agents in virtual environments
Source: Sci Rep. 2023 Sep 12;13:13914. doi: 10.1038/s41598-023-41042-3 (PMC10497614; doi:10.1038/s41598-023-41042-3)
Supplement: Supplementary file 1 — Supplementary Information 1. [file 41598_2023_41042_MOESM1_ESM.docx]

**Supplementary Information**

**Supplementary video legends**

**Supplementary video 1.** Stimuli presented in the experiment for each condition.
